# The Collagen Chaperone HSP47 Is a New Interactor of APP that Affects the Levels of Extracellular Beta-Amyloid Peptides

**DOI:** 10.1371/journal.pone.0022370

**Published:** 2011-07-28

**Authors:** Federico T. Bianchi, Paola Camera, Ugo Ala, Daniele Imperiale, Antonio Migheli, Enrica Boda, Filippo Tempia, Gaia Berto, Ylenia Bosio, Salvatore Oddo, Frank M. LaFerla, Stefano Taraglio, Carlos G. Dotti, Ferdinando Di Cunto

**Affiliations:** 1 Department of Genetics, Biology and Biochemistry, Molecular Biotechnology Center, University of Torino, Torino, Italy; 2 Centro DOMP, Azienda Ospedaliera ASL-TO2, Torino, Italy; 3 Department of Neurosciences, University of Torino, Torino, Italy; 4 Department of Physiology, University of Texas Health Science Center, San Antonio, Texas, United States of America; 5 Department of Neurobiology and Behavior, Institute for Memory Impairments and Neurological Disorders, University of California Irvine, Irvine, California, United States of America; 6 VIB Department of Molecular and Developmental Genetics and Katholieke Universiteit Leuven, Department of Human Genetics, Leuven, Belgium; Boston University, United States of America

## Abstract

Alzheimer disease (AD) is a neurodegenerative disorder characterized by progressive decline of cognitive function that represents one of the most dramatic medical challenges for the aging population. Aβ peptides, generated by processing of the Amyloid Precursor Protein (APP), are thought to play a central role in the pathogenesis of AD. However, the network of physical and functional interactions that may affect their production and deposition is still poorly understood. The use of a bioinformatic approach based on human/mouse conserved coexpression allowed us to identify a group of genes that display an expression profile strongly correlated with APP. Among the most prominent candidates, we investigated whether the collagen chaperone HSP47 could be functionally correlated with APP. We found that HSP47 accumulates in amyloid deposits of two different mouse models and of some AD patients, is capable to physically interact with APP and can be relocalized by APP overexpression. Notably, we found that it is possible to reduce the levels of secreted Aβ peptides by reducing the expression of HSP47 or by interfering with its activity via chemical inhibitors. Our data unveil HSP47 as a new functional interactor of APP and imply it as a potential target for preventing the formation and/or growth amyloid plaques.

## Introduction

Alzheimer's disease (AD) is the most common neurodegenerative disorder, with a prevalence of approximately 2% in developed countries. The risk of developing this disorder increases dramatically in individuals beyond the age of 70 and it is predicted that the incidence of AD will rise threefold within the next 50 years, hence representing an outstanding social problem [1]. From the neuropathologic point of view, AD is characterized by progressive loss of neurons and synapses, intracellular neurofibrillary tangles composed of hyperphosphorylated Tau protein, extracellular deposition of β-amyloid substance on senile plaques (SP) and cerebral amyloid angiopathy [2], [3]. The main constituents of SP are Aβ-peptides, which are generated from β-amyloid precursor protein (APP) by sequential proteolytic cleavages, mediated by β- and γ-secretases. An alternative non-amyloidogenic α-secretase cleavage cuts APP in the middle of the Aβregion [4], [5]. Although APP is a ubiquitous type I transmembrane glycoprotein [4], alternative splicing can generate at least three main isoforms, characterized by important differences in their expression pattern. The short variant, known as APP695, is the most abundant isoform in mature brain, while longer isoforms, containing a Kunitz protease inhibitor (KPI) domain, such as the APP770, are the main variants expressed in the other tissues and during brain development [6], [7]. Nevertheless, KPI-positive APP isoforms could be important in brain under abnormal conditions, since their levels are significantly increased after traumatic injury [8], after seizures [9] and in AD patients [10].

Despite the large body of knowledge accumulated in the last two decades on APP proteins, some fundamental issues about their physiological role and about their cleavage pathways remain to be fully elucidated. However, structural and functional evidences indicate that APP and APP-like proteins may function as adhesion-receptors and signal transduction molecules during cell migration [11]–[13], neurite outgrowth, dendritic arborization and synaptogenesis [14]–[16]. A full definition of the complex network of physical and functional interactions that involve APP is crucial for understanding its normal and abnormal functions and for identifying novel potential targets for therapeutic intervention. Although many players have been already identified by biochemical approaches, the picture is probably still very incomplete.

The availability of massive sets of microarray data from different species offers a unique opportunity to analyze gene function on a global scale, based on the principle that genes cooperating to the same biological functions tend to be significantly coexpressed [17]. In this report, we used a conserved coexpression approach to identify new putative functional partners of APP. We defined a gene signature strongly enriched for molecules previously implicated with APP function and/or with AD. Among these genes, we concentrated on HSP47, a serine protein inhibitor (SERPIN) well known for its collagen-chaperone activity [18]. We found that HSP47 is capable to physically interact with APP and that its intracellular localization pattern is sensitive to APP levels. Moreover, we show that HSP47 is enriched in amyloid plaques, in two different mouse models of AD and in some AD patients. Finally we show that HSP47 knockdown or pharmacological inhibition reduces the levels of extracellular Aβ peptides released by cell lines expressing normal or mutant APP, as well as by primary neuronal cultures. Our results imply that HSP47 could be a novel target for reducing Aβ levels in vivo.

## Materials and Methods

### Coexpression analysis

The list of mammalian genes showing conserved coexpression with APP was obtained from the dataset previously described [19], using essentially the same procedures, with minor modifications. Briefly, we used all the ratiometric datasets available in the Stanford Microarray Database (SMD) [20] on 01-01-2004 for human (2803 experiments, 74588 probes, comprising 6 independent APP probes) and for mouse (145 experiments, 37521 probes, comprising 6 independent APP probes). For every probe, we calculated the Pearson correlation coefficient (r_1_) with all other probes (r_x_), and ranked the probes according to the values of this index. A directed edge was established from r_1_ to r_x_ if r_x_ fell within the top 1% rows in terms of correlation with r_1_.

These directed networks were then converted into undirected networks by mapping the probes to the corresponding Entrez Gene identifiers. The list of genes whose expression is strictly co-modulated with APP is defined as the ensemble of the first-level neighbors. We then searched all the 36 possible combinations of the obtained single-species lists for the presence of orthologous genes, defined on the basis of the Homologene database, resulting in the production of 36 conserved-coexpression lists. The final list of candidates was obtained by retaining only those genes that were found in 7 conserved coexpression lists, thus ensuring that they occurred in at least 2 independent single-species coexpression lists of each organism. The Gene Ontology keyword overrepresentation was performed according to standard procedures [21].

### Cell culture

HEK293 cells stably expressing human APP carrying the Swedish mutation [22] (a kind gift from Bart De Strooper, Katolike Universite Leuven) 293 cells (ATCC) and HeLa cells (ATCC) were grown in Dulbecco's modified Eagle's Medium (DMEM) supplemented with 10% fetal calf serum (FCS). Sy5y (ATCC) cells were grown in the same medium plus 5% horse serum. Hippocampal neuronal cultures were prepared from embryonic day (E) 18 rat brains as described [23]. Rat primary cortical neurons cultures were prepared from cortices of E18 rat brains, using the protocol described in [23] with minor modifications.

### Ethics

The human sections used for this study correspond to autoptic archive material not subjected to the acquisition of an informed consent. The “Centro DOMP”, that performed the analysis of the human samples, is a diagnostic structure operating under the control of the Ethic Committe: “Comitato Etico dell'ASL-TO2, Ospedale Maria Vittoria, Torino”.

For the preparation of primary hippocampal neurons, pregnant rats and embryos were sacrificed conforming to the Italian laws on animal experimentation and under the supervision of the veterinary service of our animal facility. The corresponding experimental protocols have been approved by the Italian Ministry of Health, Department of Public Veterinary Health with approval number 22/2007-A, released on 03/14/2007.

### Antibodies

We used the following antibodies: rabbit polyclonal anti-APP C-term (Sigma); rabbit polyclonal anti-Aβ 37–42 (AB5306, Covance); mouse monoclonal anti-Aβ 1–16 (6E10, Covance); mouse monoclonal anti-HSP47 (Stressgen); rabbit polyclonal anti-Tau (ab8763 Abcam); goat polyclonal anti-Rpn2 (Santa Cruz biotechnology); rabbit polyclonal anti-GFP (Abcam); mouse monoclonal anti-MYC 9E10 (Sigma); mouse monoclonal anti βtubulin (Sigma); mouse monoclonal anti-Vinculin (Transduction Laboratory).

### Immunoblotting and immunoprecipitation

Cells were extracted with lysis buffer (1% TritonX-100, 120 mM sodium chloride, 50 mM Tris-HCl pH 7.5), protease inhibitors mixture (Roche) and 1 mM phenylmethylsulfoniyl fluoride (PMSF). For the immunoprecipitations (IPs), antibodies and protein-G-sepharose beads (Amersham, Uppsala, Sweden) were added to cleared lysates and incubated overnight at 4°C. Pellets were washed six times with lysis buffer and analysed by SDS-PAGE. For the immunoblotting, clarified extracts or IPs were analyzed by reducing SDS-PAGE and transferred to PVDF filters, which were then incubated with the indicated antibodies and developed using ECL system (Amersham Biosciences).

### In vivo cross-linking

To cross-link physically interacting proteins, cells were first exposed to DSP (Calbiochem cat#322133) at the concentration of 200 µg/ml in PBS for 10 min at room temperature, then washed twice with PBS prior to lysis. The lysates were then immunoprecipitated using Dynabeads protein G (Invitrogen), according to the manufacturers' specifications, with anti-MBP, anti-Hsp47 or anti-Aβ (6E10) monoclonal antibodies.

### Immunofluorescence microscopy and immunohistochemistry

Human sections were obtained from paraffin-embedded blocks of autoptic material derived from patients initially suspected to have prion diseases and then diagnosed to be affected by Alzheimer's disease or other disorders. Sections from paraffin-embedded blocks of brain obtained from APPPS1 transgenic mice and their littermate controls were collected onto collagen coated slides and stained using the indicated antibodies. Cells and slices were fixed with 4% paraformaldehyde in PBS and permeabilized with 0.1% TritonX-100. Immunofluorescence was performed using the indicated antibodies, followed by incubation with appropriate Alexa-Conjugated secondary reagents (Molecular Probes). Nuclei were stained with Hoescht (Sigma). All the samples were examined using Apotome or confocal imaging systems (Zeiss). For immunohistochemistry, all the reactions were performed on an automated immunostaining system (Leica). Omission of the primary Ab or substitution with an unrelated rabbit serum served as negative control.

### Real-time analysis of gene expression

6 weeks-old male FVB mice were injected intraperitoneally using 30 mg/Kg Kainic Acid monohydrated (Sigma) or with same volume of vehicle only (PBS). Seizures were observed on average one hour after the injections. 24 hours later the mice were sacrificed, hippocampi were dissected and total RNA was extracted using TRIZOL reagent (Invitrogen). Primary rat cortical neurons were transfected with plasmids or treated with compound IV. After 72 h from the medium change cells were lysed and total RNA was extracted using TRIZOL reagent (Invitrogen).

1 µg of total RNA was retrotranscribed to cDNA with random primers (New England Biolabs) using the M-MLV Reverse Transcriptase (Invitrogen) according to manufacturer specifications. Multiplex Real-Time PCR was performed on an AB 7300 station using APPlied Biosystems TaqMan Gene Expression Assays for the targets Col1a1 (GeneID: 12842), Col6a2 (GeneID: 12834), Col18a1 (GeneID: 12822), mouse HSP47 (GeneID: 12406), rat HSP47 (Gene ID: 29345), Gapdh (Gene ID: 24383), Itgb1 (GeneID: 16412), Clu (GeneID: 12759), Lrp1 (GeneID: 16971), cav1 (GeneID: 12389), Anxa5 (GeneID: 11747). For differential expression of APP695 and APP770 we generated custom differential primers: APP695 Fw = GATGGCGGTGAAGACAAAGT; APP695 Rev = CATCAGCTTCCTCTTCCTCAA; APP770 Fw = CACCACCACAACCACCACT; APP770 Rev = GGCTTGTTCAGAGCACACCT.

Real Time analysis were performed using eukaryotic 18S rRNA (APPlied Biosystems, 4319413E) for mouse samples and Gapdh for rat derived coltures as endogenous control to normalize targets expression level. Relative quantification of gene expression level was obtained using the SDS RQ Study software (Applied Biosystems). All the experiments were performed according to standard guidelines for use of research animals.

### Transfections, RNAi and treatment with the HSP47 inhibitor

For cell lines, cells were plated at a density of 10^5^ cells per well in a 24 well plate and transfected using Fugene (Mirus) or Effectene Transfection Reagent (Qiagen) for HeLa or for APPsw cells, respectively, according to the manufacturers' specifications.

Rat primary cortical neurons were electroporated before plating using Amaxa Rat Neuron Nucleofector Kit (Lonza), according to the manufacturers' specifications, and plated at a density of 2*10^6^ in 3 cm dishes.

The lentiviral shRNA constructs were obtained by Open Biosystems (RHS1764-9691844 = anti-HSP47; RHS1764-9700081 = control). shRNA constructs for the nucleofection of cortical neurons were obtained by Open Biosystems (TRCN0000008532 = Mismatched control, RCN0000008531 = R1, TRCN0000008534 = R2).

The HSP47 construct was obtained by cloning in the pcDNA plasmid (Invitrogen) the mouse HSP47 sequence (GeneBank: NM_009825.2), obtained by RT-PCR. A MYC epitope tag was inserted before the RDEL sequence. The YFP-APP expression plasmid was previously described (Kaether et al 2000).

The HSP47 inhibitor compound IV was obtained from Calbiochem (code 385874). Compounds I and III were obtained from Ryan Scientific (codes RH01393 and RF03420, respectively). In most cases cells were incubated with the inhibitor for 24 hours. Longer incubation times were used for Sy5y (48 hours) cells and for cortical neurons (72 hours).

### ELISA analysis of Aβ40 and Aβ42 levels and cell vitality

For cell lines producing wild type and/or mutated human APP, cells were plated at a density of 100000 cells per well in a 24 well plate. After the indicated treatments and times, the incubation supernatants were collected and analyzed using an Aβ40/42 High Sensitivity Kit (The Genetic Company) according to the manufacturer's specifications. Primary rat cortical neurons were plated at a density of 2*10^6^ cells in 3 cm diameter dishes. After the indicated treatments and times, the incubation supernatants were collected and analyzed using an Human/Rat βAmyloid (40) Elisa Kit Wako II (Wako), according to the manufacturer's specifications. Cells vitality was measured by Crystal Violet staining [24].

To analyze Intracellular Aβ, after the collection of the supernatant, APPsw cells were washed twice, harvested in ice cold PBS, and centrifuged. Pellets were lysed with lysis buffer (1% TritonX-100, 120 mM sodium chloride, 50 mM Tris-HCl pH 7.5), protease inhibitors mixture (Roche) and 1 mM phenylmethylsulfoniyl fluoride (PMSF). The resulting lysate was centrifuged for 10 min and then the supernatant was collected and assayed for Aß as specified above.

### Statistical Analysis

All experiments were performed on a minimum of three replicates. Data are shown as mean plus standard deviation and statistical assessment of the differences was performed using 2-tailed Student's t test.

## Results

### Identification of new potential partners of APP by conserved coexpression analysis

To obtain more insight about the physiological functions of APP and to provide a high-confidence group of new possible functional partners for this protein, we resorted to a conserved-coexpression approach [19]. To this aim, we analyzed all the available human and mouse microarray experiments deposited in the SMD, which included data from 6 human and 6 mouse independent APP probes, covering partially overlapping sets of experimental conditions. For every probe, we generated a ranked list of genes showing the highest coexpression with APP, as previously described [19]. We then performed pair-wise comparisons of the entire human and mouse results, obtaining 36 conserved-coexpression lists. The final list of candidate APP partners (Table S1, 137 genes) was obtained by selecting only the genes found in at least two independent APP coexpression lists from each organism.

Since the identification of our candidates was completely independent from a priori knowledge on APP or AD, to validate our approach we evaluated how many candidates were already linked to APP and/or AD by experimental evidence. Interestingly, for 36 candidates (>25%) it was possible to find at least one PubMed abstract in which such relationship was established (Table S1). In particular, 13 genes encode for modulators of APP metabolism or Aβ deposition, 12 are overexpressed in AD or encode for proteins enriched in AD lesions, 9 are known mediators of APP- and/or Aβ-induced downstream effects, 2 encode for proteins that colocalize with APP or affect its localization (see details in Table S1). Notably, the most well characterized partners and modulators of APP were not detected at the very stringent cutoff that we used. However, we found that Nicastrin (present in 3 lists), BRI2 (2 lists), FE65 (2 lists) and BACE1 (1 list), display a significant conserved coexpression with APP. To obtain a more systematic validation, we asked whether the candidates were significantly enriched for previously identified APP-binding proteins. Strikingly, we found that 10 of them were contained in the list of 104 APP interactors reported by the Human Protein Reference Database (HPRD) [25] (p = 6.2·E-9, exact Fisher test). Moreover, 7 of them had been genetically associated to AD, as deduced by the Alzgene database [26].

Since it has been previously shown that genes characterized by strongly conserved coexpression display very similar functional properties [17], we performed a systematic analysis of the functional annotation of our candidates, according to the Gene Ontology (GO) vocabulary [27]. The keywords displaying the most significant enrichment (Table 1) are fully consistent with the reported functions of APP. Indeed, they independently support the idea that APP plays a prominent role in cell-to-matrix interaction [11], in calcium homeostasis [28], in cell survival [29] and in neurogenesis [30].

**Table 1:** Gene ontology annotation of the candidate APP partners.

GO-keyword	Number	Percentage	Nominalp-value
Collagen	8	5.80	2.63E-10
Extracellular matrix	15	10.87	5.37E-09
Cell adhesion	20	14.49	7.13E-08
Calcium ion binding	22	15.94	8.31E-08
Apoptosis	18	13.04	8.50E-06
Integrin-mediated signaling pathway	6	4.35	3.21E-06
Neurogenesis	10	7.25	1.46E-05

Analysis of the GO functional annotation of the 137 candidate APP partners identified by human/mouse conserved coexpression analysis of the SMD. The reported keywords displayed statistically significant enrichment even after considering multiple testing (Benjamini correction, corrected p-value<0.01).The number and the percentage of genes annotated to the corresponding keyword are indicated.

The keywords showing the most striking enrichment were related to collagen biogenesis. This result is particularly interesting, because APP can physically interact with many different collagen chains [31]–[33] and because collagen XVIII [34] and fragments of collagen XXV [33] have been previously found in amyloid plaques. Therefore, the result of our bioinformatic screen suggests the interesting possibility that APP, or at least one of its isoforms, could be part of a transcriptional module activated in the central nervous system (CNS) only under specific conditions.

Since the gene expression dataset that we used for our screen does not contain many CNS-derived samples [19], to directly validate in the mammalian brain the coexpression between APP isoforms and the identified collagen-related proteins, we resorted to a mouse model of kainate-induced seizures [9]. Indeed, it has been shown that in this model KPI-positive isoforms of APP are induced, while APP695 levels are not affected [9].

We probed by real time RT-PCR the expression of three collagen chains and of HSP47, which is known to affect the maturation of many collagens by its chaperone activity [18]. Moreover, we tested some of the molecules previously correlated with APP or Aβand identified also by our screen.

**Figure 1:**
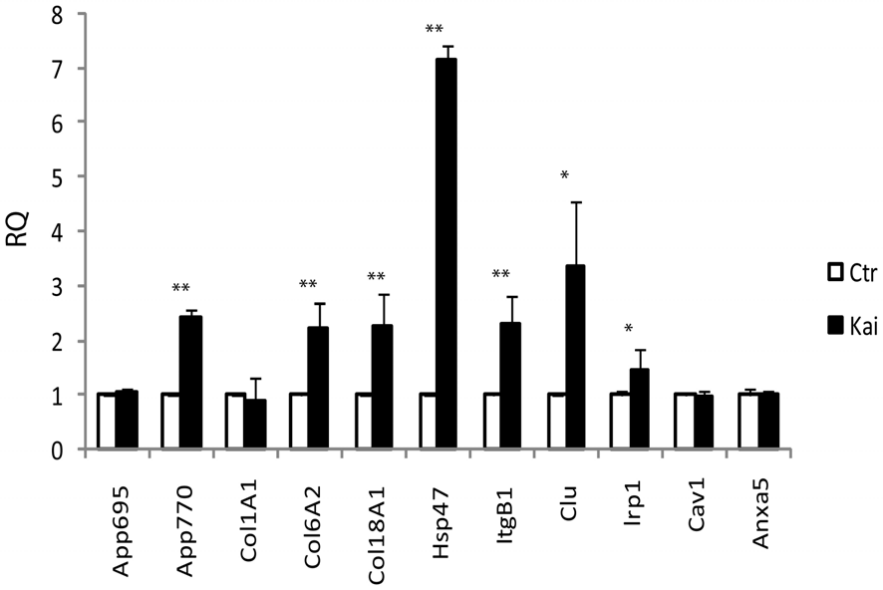
Gene signature validation in a kainate-induced seizure mouse model. 8-months old mice were treated with vehicle only (Ctr) or 30 mg/kg kainate (Kai). 24 hours after treatment, total RNA was extracted from hippocampi and subjected to real-time RT-PCR using primers specific for each of the indicated genes. Values were normalized to 18S gene expression and expressed as relative quantity (RQ) of the controls. * = p<0.05; ** = p<0.01 (two tails Student T-Test).

As expected, the hippocampus of treated mice showed a significant induction of APP770 mRNA, versus stable levels of the APP695 isoform (Fig. 1). Interestingly, most of the analyzed genes were positively regulated in this setting, with HSP47 showing the highest induction.

These results confirm that, although our original screen was performed on microarray experiments not derived from the CNS, many of the candidates may be co-regulated with KPI-positive isoforms of APP in the adult brain.

### Physical interaction and colocalization between HSP47 and APP

**Figure 2:**
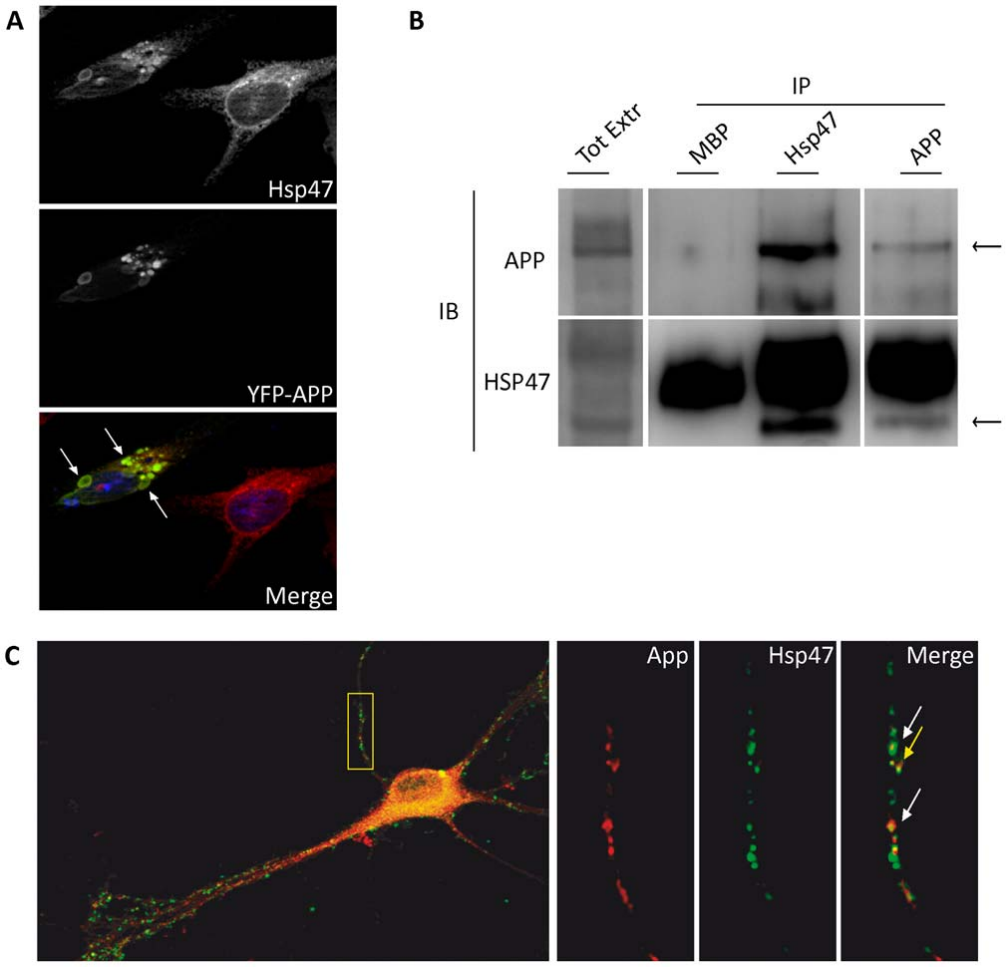
APP physically interacts with Hsp47 and affects its intracellular localization. (**A**) Recruitment of endogenous HSP47 by overexpressed APP. HeLa cells were transfected with YFP –APP and HSP47 detected by a specific anti-HSP47 antibody. Arrows indicate some of the intracellular structures showing an obvious colocalization. (**B**) Primary rat cortical neurons (4 DIV) were exposed to DSP cross linking agent and total cell lysates were immunoprecipitated with control (MBP), with anti-HSP47 or anti-APP (6E10) antibodies. The immunoprecipitates and 40 µg of the total lysate were then immunoblotted with anti APP (C-Term) or with anti HSP47. (**C**) Colocalization of HSP47 and APP in 14 DIV neurons. A high magnification field of dendrites is shown in the right panel. The signals were in general justaxposed (white arrows) and in some cases were colocalized (yellow arrows).

Among the many potential new APP partners identified by our bioinformatic survey, HSP47 emerged as an outstanding candidate for experimental validation, not only on the basis of its collagen-folder activity and co-induction with APP770 after seizures, but also because it has been previously involved in the ER stress response [35], which may play an important role in modulating Aβproduction [36]. Therefore, we asked whether HSP47 is capable to associate with APP in intact cells. To address this point we first resorted to co-immunoprecipitation assays between epitope tagged proteins, overexpressed in 293T cells. We found that, under these conditions, YFP-tagged APP was specifically associated with overexpressed HSP47 (Fig. S1A). Interestingly, immunofluorescence studies performed in HeLa cells showed that APP overexpression can dramatically affect the intracellular localization of endogenous HSP47, which was recruited to YFP-APP-positive structures in ∼50% of the transfected cells (Fig. 2A).

To address whether this interaction may occur in a more physiological context, we tried to co-immunoprecipitate the endogenous proteins from untransfected HeLa cells. However, although this cell line expresses robust levels of both HSP47 and APP, the two proteins could not be immunoprecipitated under standard conditions. A possible explanation for this discrepancy could be that the interaction between HSP47 and APP might occurr very transiently, as it is the case for many chaperone interactions, which can be detected only after chemical crosslinking [37]. Accordingly, a specific complex between HSP47 and APP was specifically immunoprecipitated from HeLa cells after treatment with the reversible crosslinker DSP [37] (Fig. S1B).

Even more importantly, using the same crosslinking protocol, the complex was immunoprecipitated also from cultured primary cortical neurons, both with anti-HSP 47 and with anti APP antibodies, but not with an unrelated antibody (Fig. 2B).

To address whether the two endogenous proteins can be found in the same cellular compartment in a physiological context, we resorted to primary rat hippocampal neurons in culture. HSP47 was clearly detectable by western blotting at all stages of the in vitro differentiation program, with a peak at 14 days in vitro (DIV - Fig. S2A), a point that in this system [38] corresponds to the beginning of synaptogenesis. Accordingly, HSP47 was also detected by immunofluorescence in both undifferentiated (data not shown) and differentiated neurons (Fig. S2B), in which it displayed a punctuate localization pattern. In agreement with the literature, this signal strongly colocalized with the rough ER marker Ribophorin II ([35], Fig. S2C), thus confirming its specificity.

Interestingly, co-staining with antibodies recognizing the C-terminal region of APP revealed that, although the two proteins tend to be in separate compartments, they are very often juxtaposed and in some cases clearly colocalized (Fig. 2C).

### HSP47 is enriched in amyloid plaques

**Figure 3:**
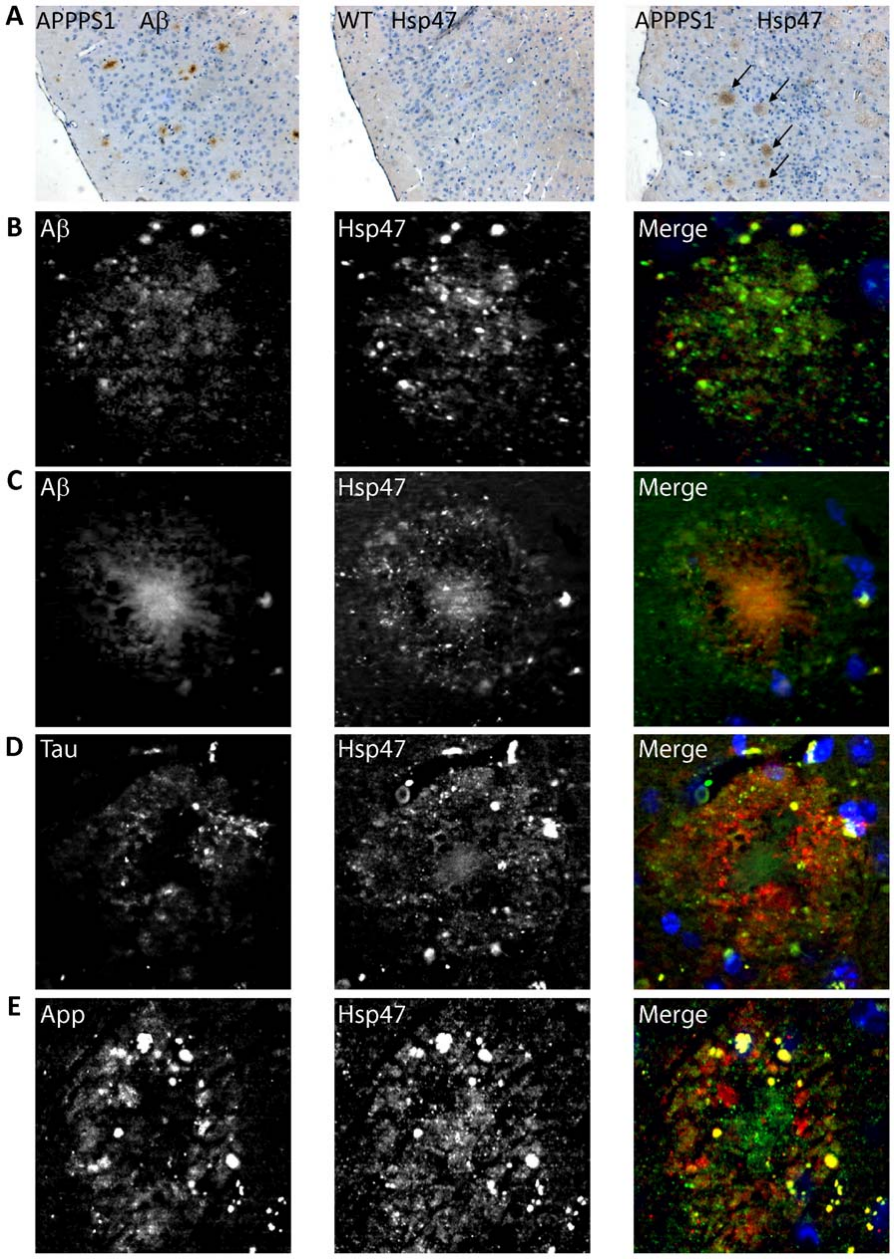
Hsp47 deposition in amyloid plaques of AD mouse models. (**A**) Immunohistochemical analysis of Aβ and HSP47 on wild type (WT) and APPPS1 transgenic mice (12 months old). Arrows indicate HSP47 positive plaques. (**B–C**) Double immunostaining of APPPS1 brain slices with HSP47 and Aβ antibodies. The diffuse plaques (B) show a marked colocalization of the two signals. In dense core plaques (C), the HSP47 antibody stained the Aβ core and a surrounding coronal area. (**D–E**) Dense core plaques double-stained with HSP47 and Tau (D) or APP C-terminal (E) antibodies.

**Figure 4:**
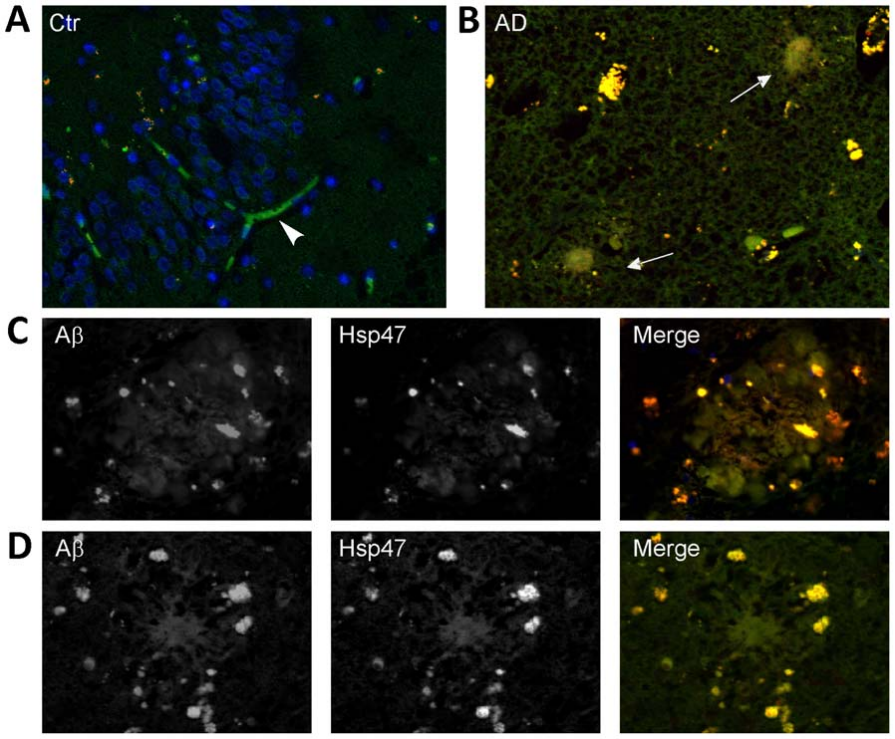
Hsp47 is enriched in amyloid plaques of AD patients. (**A**) Immunofluorescence analysis of HSP47 distribution (green) in normal human brain (Ctr). The arrowhead indicates a positive vessel. (**B**) Analysis of the distribution of HSP47 (green) and Aβ (red) in one of the III–IV stage AD cases. Arrows indicate two HSP47-positive amyloid plaques. (**C–D**) High magnification fields of AD brain sections analyzed as in B, showing diffuse (C) and dense-core (D) HSP47-positive plaques.

We then asked whether the expression and/or distribution of HSP47 are altered in presence of abnormal APP cleavage. To this aim, we analysed 12-month-old APPPS1 mice, which co-express KM670/671NL mutated amyloid precursor protein and L166P mutated Presenilin 1 under the control of the neuron-specific Thy1 promoter [39]. APPPS1 mice show dramatic deposition of Aβ starting at 6–8 weeks in the neocortex, becoming widespread in the whole CNS at 8 months ([39] and Fig. 3A). These Aβ deposits appear both as small, congophilic dense-core amyloid plaques and as diffuse plaques. By immunohistochemistry, we detected high levels of HSP47 in the blood vessels and in the choroid plexus of both control and APPPS1 brains (data not shown), as expected from the literature [40]. However, in addition to the vascular positivity, APPPS1 mice showed a clear signal in amyloid plaques (Fig. 3A). Interestingly, this positivity was already detectable in the small plaques of young mice (Fig. S3), thus suggesting that HSP47 deposition occurs throughout the process of plaques formation, starting from its earliest stages. No enrichment was detected under the same conditions using antibodies recognizing BiP, a well established marker of the ER (Fig. S4). Double immunostaining showed that HSP47 is enriched in all types of amyloid deposits, with a peculiar distribution compared to that of Aβ. In particular, in plaques formed by diffuse Aβ deposition, HSP47 and Aβ immunostaining showed the same overall distribution and a very high degree of colocalization (Fig. 3B). Moreover, in dense core plaques, anti-HSP47 antibodies stained the amyloid core, overlapping with anti-Aβ staining, but showed also a distinct punctuate pattern in an Aβ-negative region surrounding the core, that has been previously shown to contain dystrophic neurites [39]. Accordingly, these areas were specifically labeled by anti-Tau and anti-APP antibodies, whose signals significantly overlapped with the HSP47 staining (Fig. 3C–D). A similar HSP47 enrichment was clearly detectable by immunohistochemistry in the amyloid plaques produced by 3×Tg-AD transgenic mice [41], a second well-studied model of AD characterized by much less pronounced amyloidosis. This finding excluded the possibility that the results obtained with APPPS1 mice might be a non-specific effect of the dramatically increased Aβ levels that characterize this particular transgenic line. By western blot analysis, we could not detect significant differences in the total levels of HSP47 in APPPS1 mice versus controls (data not shown), most likely because of the relatively stronger vascular expression of the protein. To evaluate whether the HSP47 enrichment detected in the mouse models could be relevant to human AD pathology, we analyzed by immunofluorescence microscopy brain sections from four AD patients, including one Braak & Braak [42] stage V–VI case and three III–IV stage cases, alongside two normal controls. As expected, in the normal samples HSP47 positivity was mainly restricted to vascular structures (Fig. 4A). A similar pattern was observed in two of the III–IV stage patients, which did not show any HSP47 enrichment in amyloid plaques (data not shown). Interestingly, in the two remaining patients, a clear positivity was detected in some of the diffuse amyloid plaques and in the majority of the dense core plaques (Fig. 4B). Remarkably, in the positive plaques, the distribution of HSP47 and its colocalizazion with Aβ (Fig. 4C–D) were essentially identical to those observed in APPPS1 mice (Fig. 3). Altogether, the above results indicate that HSP47 may be a novel component of AD-related amyloid plaques.

### HSP47 modulates the levels of extracellular Aβ peptides

**Figure 5:**
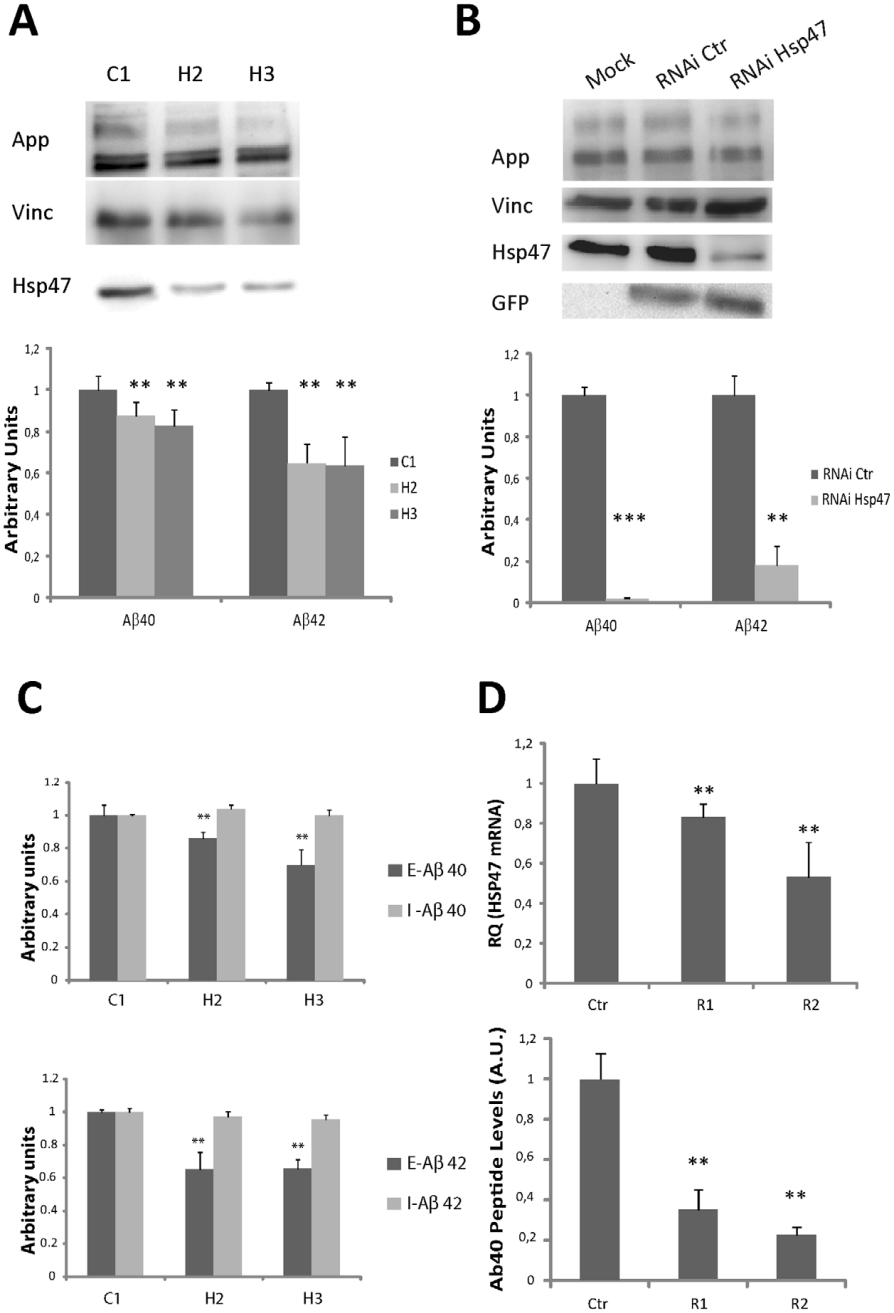
HSP47 modulates the levels of extracellular Abeta peptides. (**A**) APPsw cells were transiently transfected with two independent siRNA oligonucleotides (H2 and H3) designed against the human HSP47 sequence or with a mismatch control (C1). After additional 36 h in culture, total cell lysates were analyzed by western blotting (upper panel) with anti HSP47, APP and Vinculin (Vinc) antibodies. In parallel, the amount of A*β* peptide species was determined in the conditioned medium by ELISA (lower panel). (**B**) APPsw were infected with miRNA-based RNAi lentiviral vectors directed towards HSP47 or towards an unrelated sequence (RNAi Ctr), expressing also a GFP protein. Polyclonal populations were then analyzed as in (A) 36 h after the last medium change. (**C**) Extracellular (E-Aβ) and intracellular (I-Aβ) Aβ peptides were measured separately in APPsw cells treated as in panel A. (**D**) Primary rat cortical neurons were electroporated with two indipendent plKO plasmids (R1 and R2) encoding for shRNAs directed towards HSP47 or with a plKO containing a mismatched sequence (Ctr). 48 h after electroporation medium was changed and, after additional 72 hours, RNA was exctracted to evaluate Hsp47 knockdown by a Real Time PCR assay. In parallel, the amount of Aβ40 in the conditioned medium was determined by an ELISA assay directed against rodent Aβ40 (WAKO). ** = p<0.01; *** = p<0.001 (two tails Student T-Test).

The presence of HSP47 in amyloid deposits could be related to a role of this protein in amyloidogenic cleavage of APP and/or in the deposition of Aβ or it could simply reflect a secondary, bystander involvement. To discriminate between these possibilities, we decided to address whether HSP47 may modulate the levels of Aβ peptides in cellular models. We first performed HSP47 RNAi in the 293 APPsw cell line, which expresses a human APP gene carrying the Swedish mutation and produces high amount of Aβpeptides [22]. We found two independent siRNA sequences capable of reducing HSP47 protein levels by approximately 50% (Fig. 5A). Interestingly, both siRNAs determined a significant reduction of secreted Aβ-40 and Aβ-42 peptides, if compared to a mismatch control (Fig. 5A). Since we could not obtain a better knockdown of HSP47 by siRNA transfection, to reduce more dramatically HSP47 levels we resorted to miRNA-based lentiviral vectors [43]. After selection, the cells transduced with an anti-HSP47 construct displayed a 90% reduction of protein levels, compared to cells infected with a control construct (Fig. 5B). Importantly, if compared to control cells, the levels of Aβ-40 and Aβ-42 peptides measurable in the conditioned medium of the HSP47 knockdown cells displayed a dramatic reduction, much more pronounced than the reduction obtained upon transient silencing (Fig. 5B). In both transiently and stably HSP47-interfered cells, APP protein levels were not affected (Fig. 5A and 5B). In addition, differently from extracellular Aβ, the levels of intracellular Aβdid not change significantly upon HSP47 RNAi (Fig. 5C). A significant reduction of Aβpeptides was also observed in HeLa cells upon transient knockdown (Fig. S5), thus excluding the possibility that the effects observed in 293sw cells are due to APP overexpression and/or to the presence of a mutated APP sequence. To address whether HSP47 may affect extracellular Aβ levels in a more physiological context, we performed a knockdown experiment in rat primary cortical neurons, by transfecting shRNA plasmids. Even in this case, in cells transfected with plasmids capable of reducing HSP47 mRNA levels, we observed a strong reduction of Aβ-40 levels (Fig. 5D).

### Chemical inhibitors of HSP47 decrease the levels of secreted Aβ peptides

**Figure 6:**
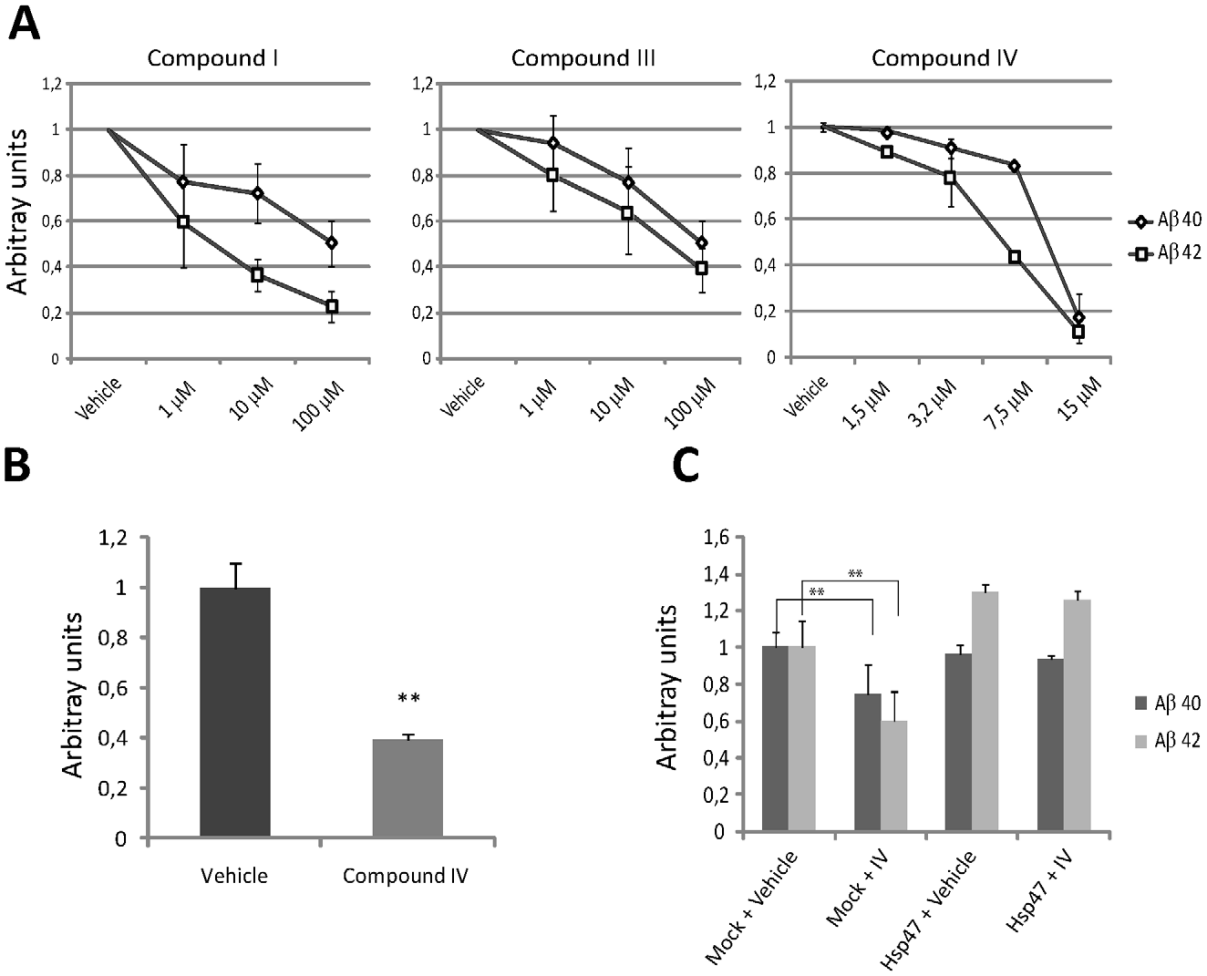
Chemical inhibition of HSP47 decreases the levels of extracellular Abeta peptides. (**A**) Dose dependent reduction of Aβ peptides levels by increasing concentrations of HSP47 inhibitors in 293 APPsw cells. The amount of Aβ peptides species in the conditioned medium of cells treated with the indicated inhibitors was determined by ELISA 24 hours after the last medium change and expressed as ratio on the vehicle control. (**B**) Primary rat cortical neurons were treated 24 h after plating with Compound IV (7.5 βµM) or Vehicle (DMSO). The amount of Aβ40 in conditioned medium was then evaluated as in Fig. 5C. (**C**) 293 APPsw cells were transiently transfected with an unrelated control plasmid (Mock) or with an HSP47 overexpression construct. 12 hours later, cells were treated with vehicle only or with 7.5 µM Compound IV (IV). The amount of Aβ peptides in the conditioned medium was determined after additional 24 hours as in (A). ** = p<0.01 (two tails Student T-Test).

Chemical inhibitors of HSP47 have been previously developed, as lead compounds potentially useful for controlling fibrosis and metastasis [44]. Therefore, in light of the above results, we wondered whether the levels of Aβ peptides in the conditioned medium could be inhibited not only by decreasing HSP47 levels, but also by modulating its biochemical activity. To address this point, we treated APPsw cells with three previously described HSP47 inhibitors [44], which are capable to affect its collagen-folding ability at low-micromolar concentrations. The HSP47 inhibitors had no detrimental effects on cell viability at concentrations as high as 100 µM (data not shown). Interestingly, all three compounds decreased the levels of Aβ peptides in the conditioned medium, in a dose-dependent manner (Fig. 6A). The molecule referred as compound IV displayed the most striking effect, because it reduced Aβ peptides by ∼90% at a 15 µM concentration (Fig. 6A). Similar effects were observed also in HeLa cells (Fig. S6), in Sy5y cells (Fig. S6) and, most importantly, in rat primary cortical neurons (Fig. 6B).

Since the HSP47 inhibitors act most likely by non-covalent binding [44] we reasoned that, if these effects were specific, they should be overcome by increasing HSP47 levels. Therefore, we measured the amount of Aβ peptides in the conditioned medium of 293sw cells transfected with an HSP47 expression construct or with a control vector, after treatment with vehicle only or with the Compound IV (7.5 µM). Interestingly, in cells overexpressing HSP47, the levels of Aβ40 were similar to the levels detected in non-tranfected cells, while Aβ42 levels were significantly increased (Fig. 6C). More importantly, in transfected cells, the levels of both peptides were not significantly affected by treatment with the compound IV (Fig. 6C). Altogether, these results indicate that HSP47 levels and activity can profoundly modify the amount of secreted Aβpeptides.

## Discussion

Most of the efforts that have been so far devoted to the identification of molecules that may affect AD pathogenesis by functionally interacting with APP were based on biochemical strategies or on genetic approaches [5]. In this study we have pursued a similar objective, using a completely different method. Our approach was based on the assumption that some of the functional APP partners may share with this molecule a very similar mRNA expression profile. On this basis, we performed a systematic search for genes that display an expression profile strongly similar to APP in both human and mouse, taking advantage of the shear amount of microarray data available for both organisms. This procedure led to the identification of 137 candidate genes. Considering that the majority of the experiments that compose the starting dataset are not derived from CNS samples [19], it could be argued that our candidates are not very likely to functionally interact with APP in brain or to play any role in Aβ deposition. This view would be consistent with the fact that a large percentage of the candidates are known to be expressed at very low levels in brain. Moreover, many of the most intensely studied physical and functional interactors of APP were not present in the list. Nevertheless, our approach is validated by the fact that more than 25% of the candidates were already linked in some way to APP or AD. In addition, we found evidence that also BACE1, Nicastrin, BRI2 and Fe65 are significantly coexpressed with APP, although this becomes evident only by slightly relaxing the inclusion cutoff. Even more strikingly, the functional annotation of the candidates is completely consistent with the available knowledge on APP, further underscoring its role in processes involving cell-to-extracellular matrix interaction. Therefore, considering that our approach is completely independent from previous literature, it is very likely that many of the new candidates will turn out to be actual functional interactors of APP.

The analysis of an experimental model of epilepsy confirmed that the coexpression between APP and many of the candidates can be detected also in CNS tissues. Moreover, this analysis suggested that, in the CNS, the expression of our candidates may correlate more with KPI-positive isoforms of APP than with the APP695 isoform. This scenario could be very significant for AD, since stressful conditions [45], [46] and the KPI-positive isoforms [10], [15], [47] have been positively correlated with Aβ deposition.

Under this perspective, we think that collagens and molecules implicated in collagen biogenesis are extremely interesting candidates. Indeed, collagens are the most enriched protein family in our list (Table 1). In addition, it was previously shown that APP can directly bind many different collagen chains [31]–[33]. Moreover, the deposition of different collagen types has been documented in epileptic brains [48] and in AD amyloid plaques [33], [34]. On this basis, but also considering its striking upregulation in the kainate-induced seizure model and its localization to the ER, we thought to address the possibility that HSP47, a master player in collagen biogenesis, might be related to APP in an AD-related context.

The first relevant finding in this direction is that HSP47 is enriched in amyloid plaques of APPPS1 [39] and of 3×Tg-AD [41] transgenic mice. Indeed, while the expression of HSP47 was very low in the parenchyma of control brains, it was easily detectable in amyloid plaques of both models, especially after formic acid pre-treatment of the sections. Importantly, we were able to detect a similar enrichment in the diffuse and dense-core plaques of some AD cases in advanced stages. Although more studies are clearly necessary to reach statistically significant conclusions on this point, the latter result suggests that HSP47 could be involved in amyloid deposition in a subset of AD patients. Thus, it will be very interesting to deeply investigate whether HSP47 enrichment correlates with some specific clinical features and/or with disease stage.

We cannot yet reach definitive conclusions about the source of the HSP47 protein deposited in amyloid plaques. The fact that we did not observe significant increase of the total amount of HSP47 would seem to exclude a mechanism based on increased expression. However, it is still possible that the deposition is due to locally increased expression, which would be hard to detect over the predominant pool of HSP47 coming from blood vessels. Alternatively, the high local concentration of HSP47 in plaques could derive exclusively from a local redistribution of the protein. In both cases, it is possible that some of the deposited HSP47 derives from neuronal cells. Indeed, primary neurons in culture express significant levels of HSP47, showing the expected subcellular distribution and a low but significant degree of colocalization with APP (Fig. 2C). Moreover, we clearly detected HSP47 in a region of the dense-core plaques that is characterized by the presence of dystrophic neurites, as demonstrated by increased tau deposition. The latter observation suggests that the levels of HSP47 could be locally increased in regions undergoing pathologic remodelling. This scenario is supported by the findings that APP physically interacts with HSP47 (Fig. 2B and S1) and that endogenous HSP47 can be recruited to intracellular compartments overexpressing APP (Fig. 2A).

Our in vitro experiments on cultured cells provide direct proof of concept that HSP47 could play a role in the extracellular accumulation of Aβpeptides. Indeed, in primary rat cortical neurons, in cells expressing normal levels of wild type APP and in cells overexpressing mutant APP, the levels of Aβ peptides in the conditioned medium were consistently reduced after HSP47 knockdown. Most likely, this effect is not due to a modulation of the APP amyloidogenic cleavage. Indeed, total APP levels were normal by western blotting. Moreover, only the levels of extracellular Aβ are decreased by HSP47 knockdown. On the basis of these results, the most likely working hypotheses are that HSP47 could affect the degradation or the solubility of Aβpeptides in the extracellular milieu. On the first line, we find very interesting that HSP47 is a predicted serine proteinase inhibitor. As such, it could interfere with the degradation of Aβmediated by plasmin [49] or by other proteases, such as the heat shock protein Omi, which can preferentially bind and destroy oligomeric Aβ [50].

On the other hand, a conceivable scenario is that HSP47 could influence the described interaction between APP and collagens or between Aβ peptides and collagens [31]–[33]. By doing so, it could modulate the deposition of an insoluble matrix containing both collagen and APP degradation products, which could also explain the enrichment detected in amyloid plaques. The finding that small molecules affecting the collagen chaperone capability of HSP47 can decrease the levels of extracellular Aβ peptides, an activity that can be rescued by HSP47 overexpression, suggests that the action of HSP47 could be actually related to collagen production. However, we cannot exclude that HSP47 may act more directly and that the inhibitors may influence an alternative activity of the protein, not impinging on collagen folding.

The latter hypothesis would be supported by the poor correlation between the activity of the tested molecules versus collagen-folding and their Aβ-reducing capability (compare Fig. 6 with [44]), even though many other factors may explain this discrepancy.

In conclusion, although much more detailed work is necessary to dissect these potential mechanisms and to analyze their possible role in plaque formation, we propose that the modulation of HSP47 activity, through small molecules, could be a novel approach to modulate in vivo the levels of soluble Aβpeptides.

## Supporting Information

Figure S1 **Coimmunoprecipitation of overexpressed and endogenous APP and HSP47 in cell lines.** (**A**) Co-immunoprecipitation of APP and HSP47. Total cell lysates from HEK293T cells either untransfected (Mock) or co-transfected with YFP-APP695 and with MYC-HSP47 or an unrelated control (MYC-CITΔN) were immunoprecipitated with anti-MYC antibodies and analyzed by immunoblotting with anti GFP (upper panel). The expression of recombinant proteins in the lysate was verified by immunoblotting, as indicated (middle and lower panels). (**B**) HeLa cells were exposed to DSP cross linking agent and total cell lysates were immunoprecipitated with control (MBP) or anti-HSP47 antibodies. The immunoprecipitates and 40 µg of the total lysate were then immunoblotted with anti APP (C-Term) or with anti HSP47.
(TIF)

Figure S2 **Expression of Hsp47 in primary hippocampal neurons.** (**A**) Total cell lysates (20 µg) of hippocampal neurons kept in culture for the indicated time (DIV = days in vitro) were analyzed by western blotting with anti-HSP47 antibodies. Beta-tubulin (βtub) antibodies were used as internal loading control. (**B**) Immunofluorescence analysis of HSP47 on 14 DIV primary hippocampal neurons. Note the punctuate staining pattern. (**C**) Colocalization of HSP47 and the rough-ER marker Ribophorin-II (Rpn2) in 14 DIV neurons. A high magnification field of dendrites is shown in the right panel. Arrows indicate some points of colocalization.
(TIF)

Figure S3 **Time course analysis of the Hsp47 deposition in amyloid plaques of AD mose models.** Hsp47 deposition in amyloid plaques is an early event occurring in two different AD mouse models. (A–C) Serial thin sections of the cortex of APPPS1 mice at 3 (A), 9 (B) and 12 months of age were stained for Hsp47 and Aβ. (D) Serial thin sections of 12 months-old 3×Tg-AD mouse brains were stained as above. Note that, in this model, the number of plaques was much lower than in APPPS1 mice of comparable age. The white arrow indicates a positive plaque. Scale bars: 200 µm (A–C); 100 µm (D).
(TIF)

Figure S4 **Specificity of HSP47 antibody staining in amyloid plaques of AD APPPS1 mouse model.** Specificity of Hsp47 enrichment in amyloid plaques of APPPS1 mice. Immunohistochemistry of cortical serial sections of 9 months old APPPS1 mice, performed with the indicated primary antibodies and with the same secondary reagents. The HSP47- positive amyloid plaques indicated by arrows are not detected by anti BiP antibodies.
(TIF)

Figure S5 **Lowering of Hsp47 in HeLa cells decreases the levels of extracellular Abeta peptides.** HeLa cells were transiently transfected with two independent siRNA oligonucleotides (h2 and h3) designed against the human HSP47 sequence or with a mismatch control (r1). After additional 36 h in culture cell viability was determined the amount of Aβ peptide species in the conditioned medium was determined by ELISA. Values are expressed as ration on the control. * = p<0.05; ** = p<0.01 (two tails Student T-Test).
(TIF)

Figure S6 **Chemical inhibition of Hsp47 in HeLa cells and Sy5y cells decreases the levels of extracellular Abeta peptides.** HeLa or Sy5y cells were treated with vehicle only or with 7.5 µM Compound IV for 24 or 48 hours, respectively. The concentration of Aβ peptides in the conditioned medium was then determined by ELISA analysis and reported as ratio on the control. * = p<0.05; ** = p<0.01; *** = p<0.001 (two tails Student T-Test).
(TIF)

Table S1 **List of candidate APP partners identified by the coexpression-based bioinformatic screen.** List of the 137 candidates identified by conserved coexpression analysis on the SMD dataset. A = colocalized with APP or affecting APP localization; B = overexpressed in AD or found in AD lesions; C = modulator of APP metabolism and of Aβ deposition; D = downstream mediator of APP or Aβ; E = APP binding partner. Asterisks indicate the genes reported to encode for APP interacting proteins in the HPRD database and the genes genetically linked to AD in the Alzgene database. The last column (N) indicates the number of APP conserved coexpression lists in which the corresponding gene was found. The genes are ranked by decreasing N.
(PDF)
