# Supplementary figures and images for: The Collagen Chaperone HSP47 Is a New Interactor of APP that Affects the Levels of Extracellular Beta-Amyloid Peptides
Source: PLoS One. 2011 Jul 28;6(7):e22370. doi: 10.1371/journal.pone.0022370 (PMC3145648; doi:10.1371/journal.pone.0022370)

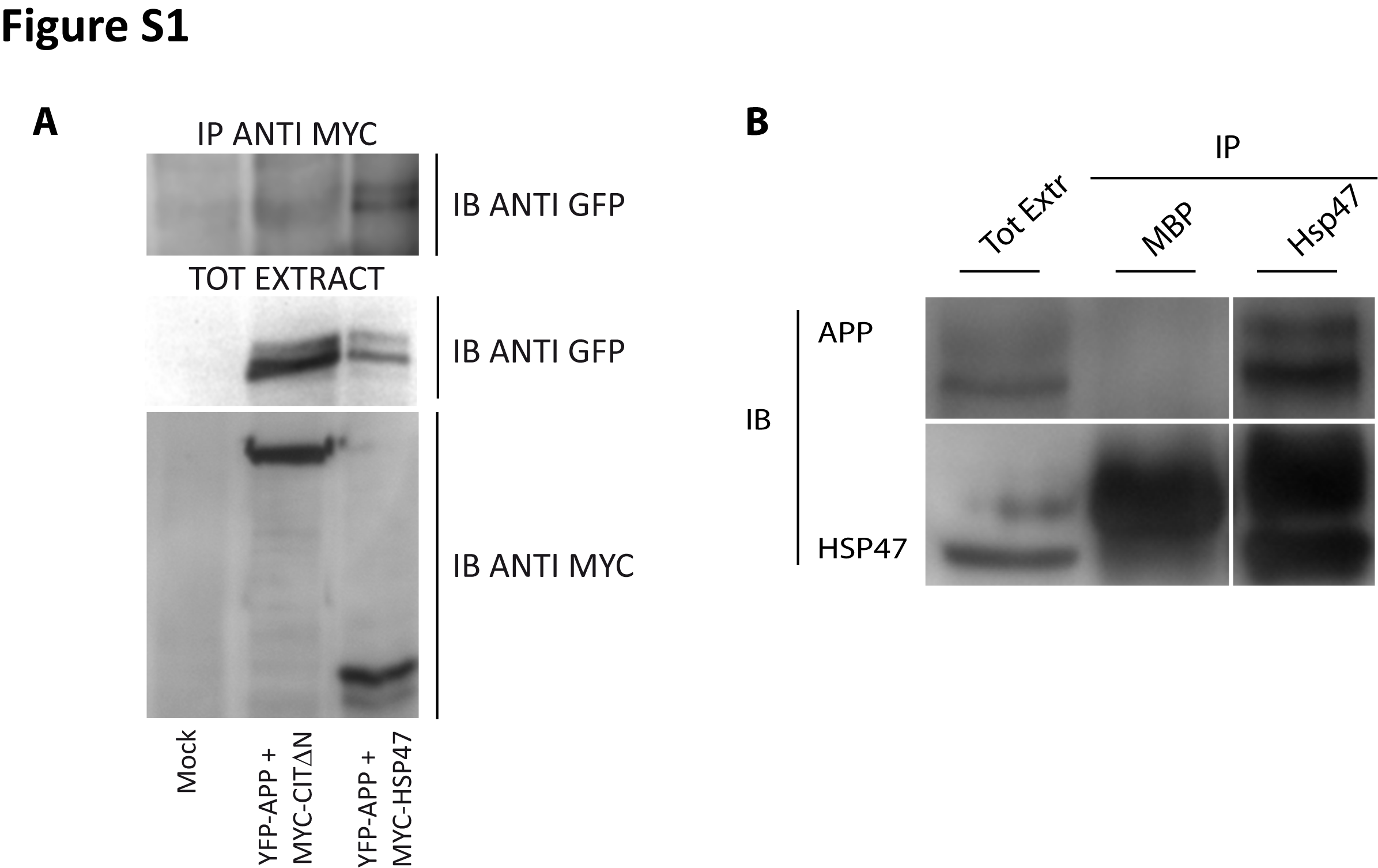

Supplement: Figure S1 — Coimmunoprecipitation of overexpressed and endogenous APP and HSP47 in cell lines. (A) Co-immunoprecipitation of APP and HSP47. Total cell lysates from HEK293T cells either untransfected (Mock) or co-transfected with YFP-APP695 and with MYC-HSP47 or an unrelated control (MYC-CITΔN) were immunoprecipitated with anti-MYC antibodies and analyzed by immunoblotting with anti GFP (upper panel). The expression of recombinant proteins in the lysate was verified by immunoblotting, as indicated (middle and lower panels). (B) HeLa cells were exposed to DSP cross linking agent and total cell lysates were immunoprecipitated with control (MBP) or anti-HSP47 antibodies. The immunoprecipitates and 40 µg of the total lysate were then immunoblotted with anti APP (C-Term) or with anti HSP47. (TIF) [file pone.0022370.s001.tif]

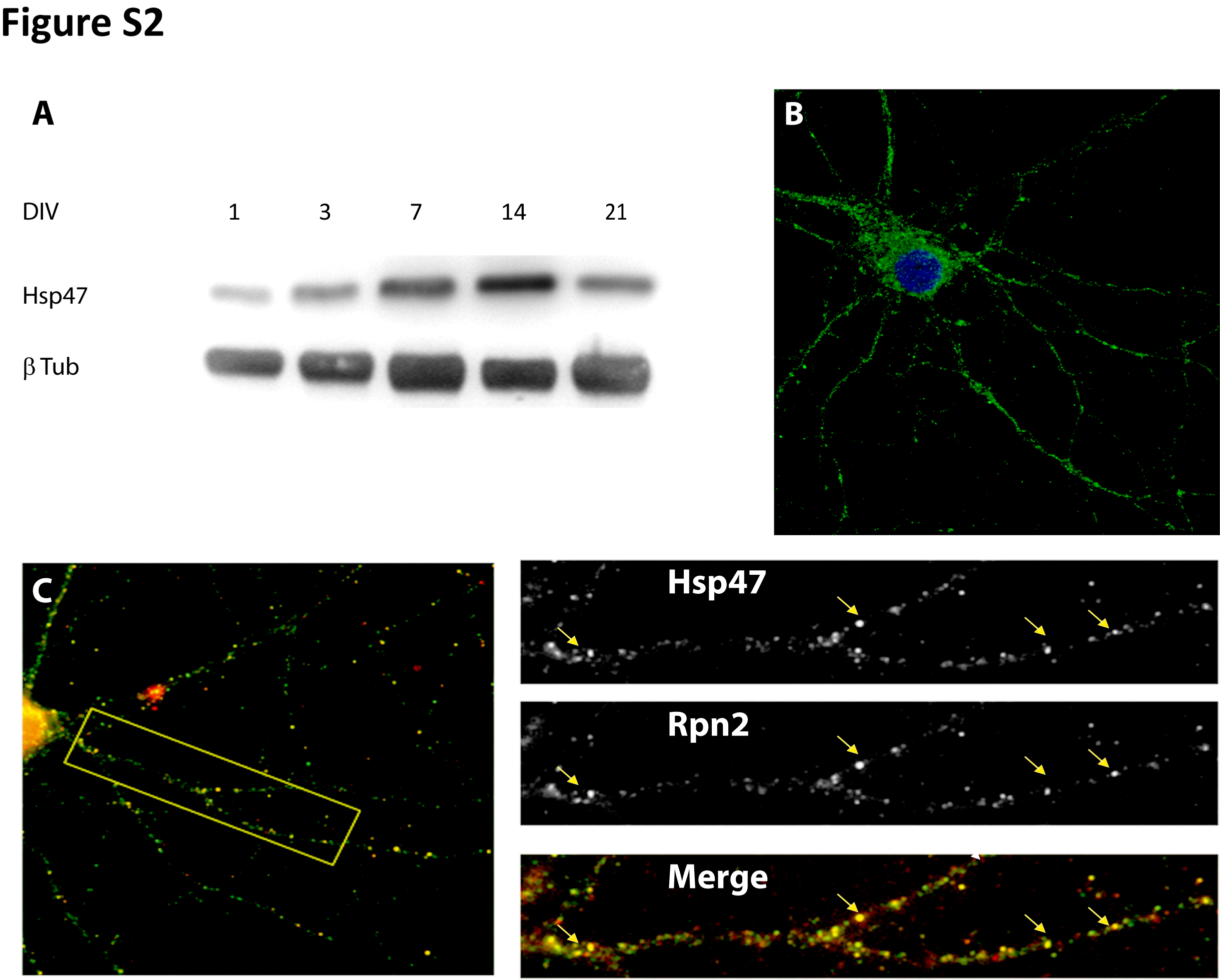

Supplement: Figure S2 — Expression of Hsp47 in primary hippocampal neurons. (A) Total cell lysates (20 µg) of hippocampal neurons kept in culture for the indicated time (DIV = days in vitro) were analyzed by western blotting with anti-HSP47 antibodies. Beta-tubulin (βtub) antibodies were used as internal loading control. (B) Immunofluorescence analysis of HSP47 on 14 DIV primary hippocampal neurons. Note the punctuate staining pattern. (C) Colocalization of HSP47 and the rough-ER marker Ribophorin-II (Rpn2) in 14 DIV neurons. A high magnification field of dendrites is shown in the right panel. Arrows indicate some points of colocalization. (TIF) [file pone.0022370.s002.tif]

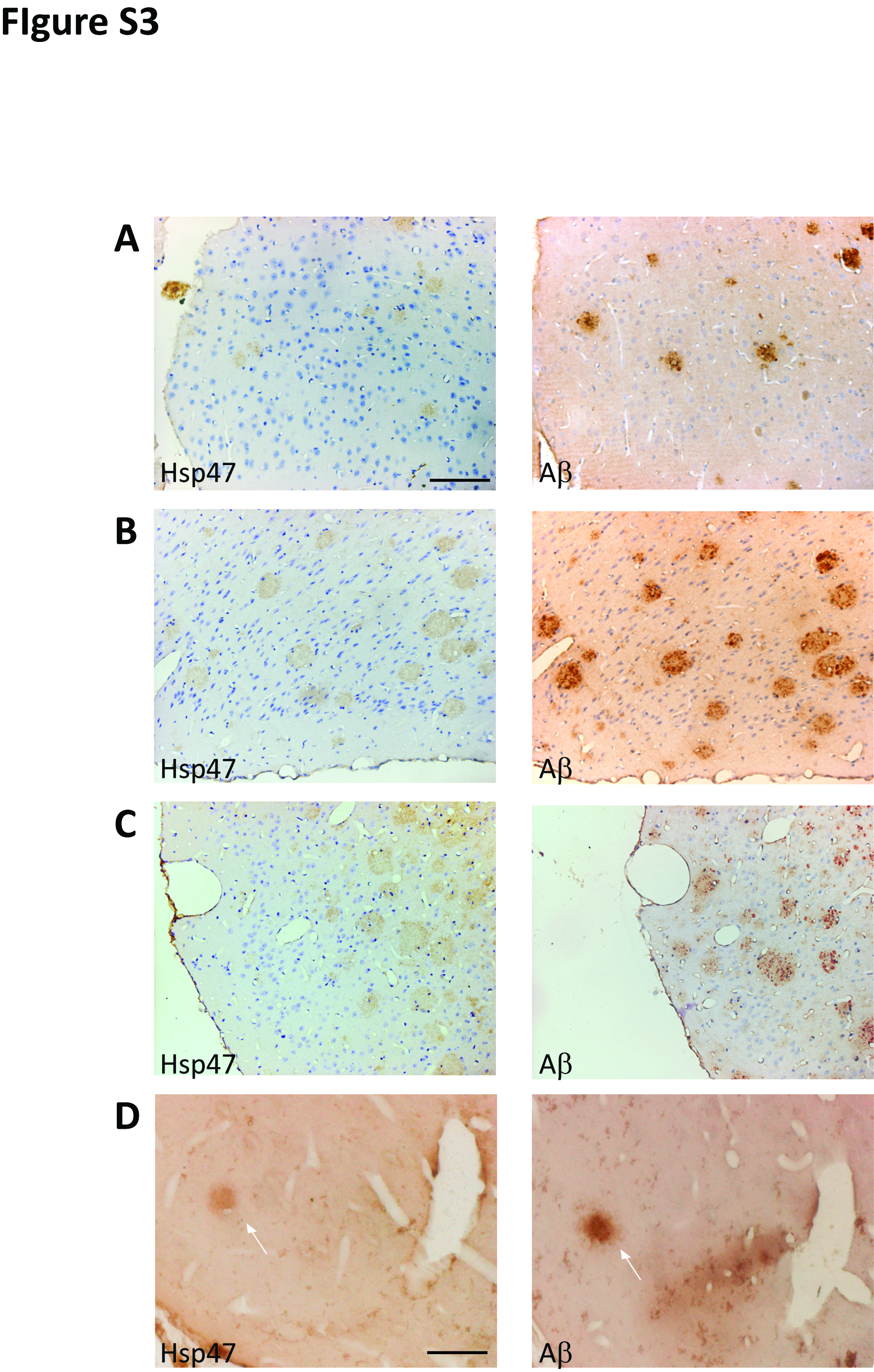

Supplement: Figure S3 — Time course analysis of the Hsp47 deposition in amyloid plaques of AD mose models. Hsp47 deposition in amyloid plaques is an early event occurring in two different AD mouse models. (A–C) Serial thin sections of the cortex of APPPS1 mice at 3 (A), 9 (B) and 12 months of age were stained for Hsp47 and Aβ. (D) Serial thin sections of 12 months-old 3×Tg-AD mouse brains were stained as above. Note that, in this model, the number of plaques was much lower than in APPPS1 mice of comparable age. The white arrow indicates a positive plaque. Scale bars: 200 µm (A–C); 100 µm (D). (TIF) [file pone.0022370.s003.tif]

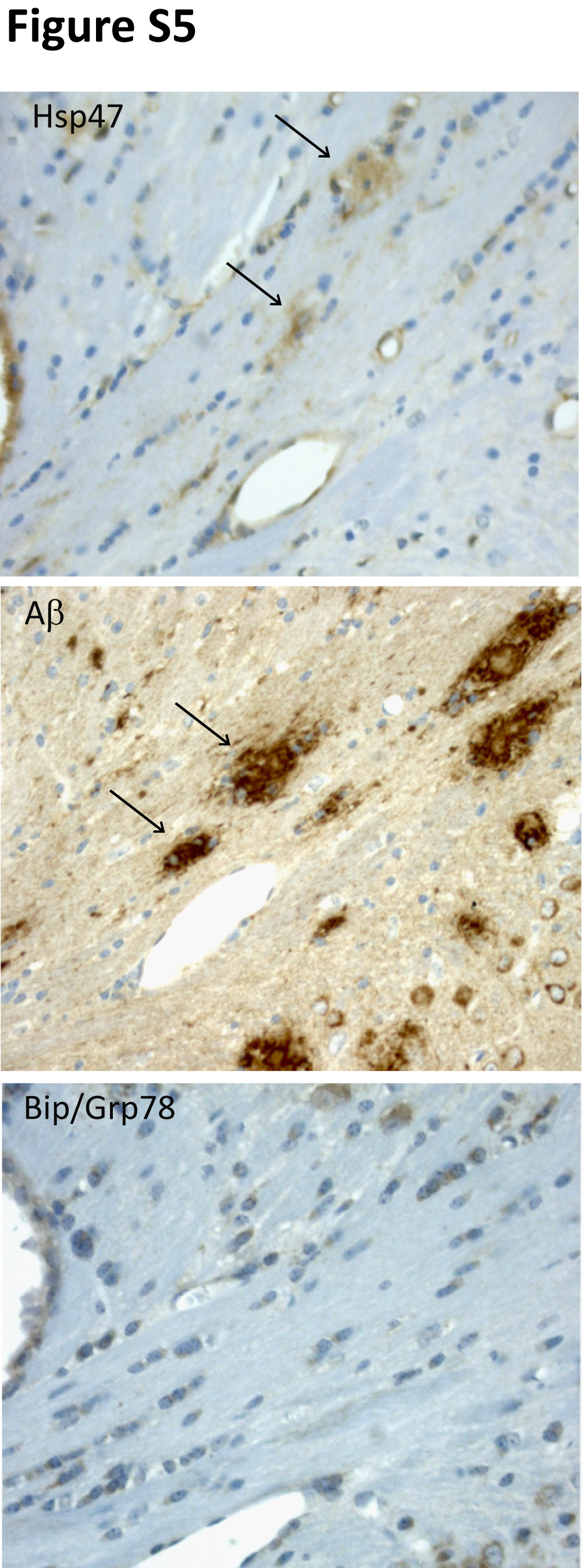

Supplement: Figure S4 — Specificity of HSP47 antibody staining in amyloid plaques of AD APPPS1 mouse model. Specificity of Hsp47 enrichment in amyloid plaques of APPPS1 mice. Immunohistochemistry of cortical serial sections of 9 months old APPPS1 mice, performed with the indicated primary antibodies and with the same secondary reagents. The HSP47- positive amyloid plaques indicated by arrows are not detected by anti BiP antibodies. (TIF) [file pone.0022370.s004.tif]

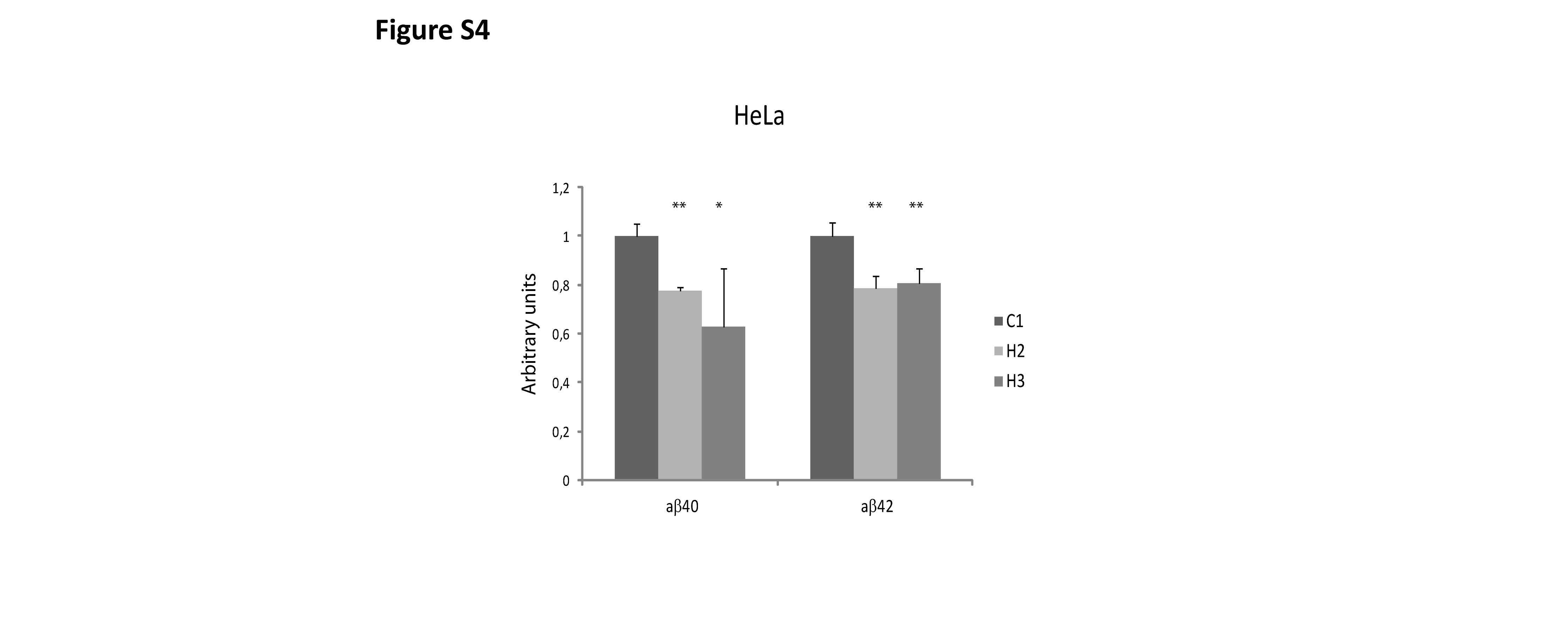

Supplement: Figure S5 — Lowering of Hsp47 in HeLa cells decreases the levels of extracellular Abeta peptides. HeLa cells were transiently transfected with two independent siRNA oligonucleotides (h2 and h3) designed against the human HSP47 sequence or with a mismatch control (r1). After additional 36 h in culture cell viability was determined the amount of Aβ peptide species in the conditioned medium was determined by ELISA. Values are expressed as ration on the control. * = p<0.05; ** = p<0.01 (two tails Student T-Test). (TIF) [file pone.0022370.s005.tif]

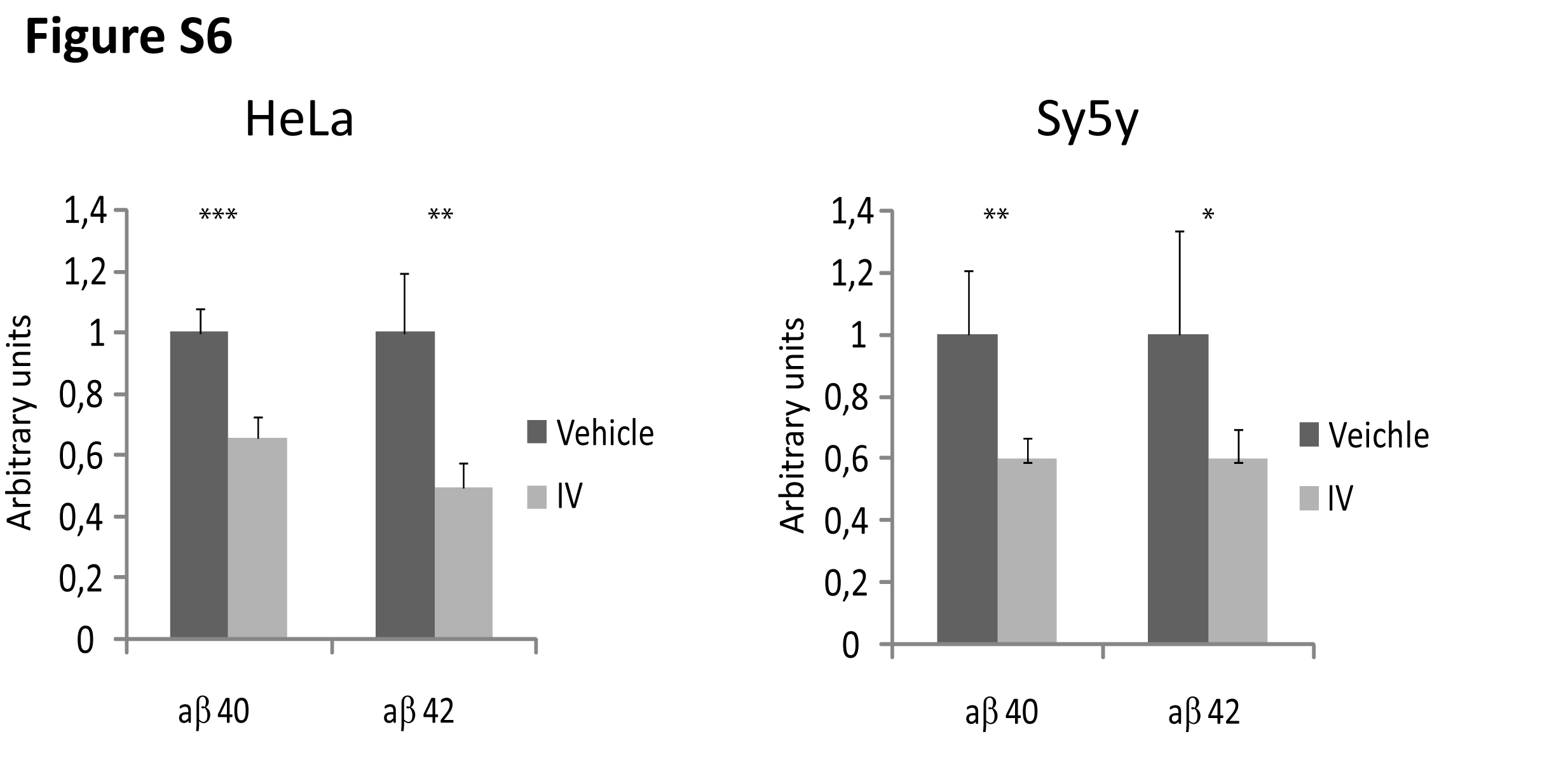

Supplement: Figure S6 — Chemical inhibition of Hsp47 in HeLa cells and Sy5y cells decreases the levels of extracellular Abeta peptides. HeLa or Sy5y cells were treated with vehicle only or with 7.5 µM Compound IV for 24 or 48 hours, respectively. The concentration of Aβ peptides in the conditioned medium was then determined by ELISA analysis and reported as ratio on the control. * = p<0.05; ** = p<0.01; *** = p<0.001 (two tails Student T-Test). (TIF) [file pone.0022370.s006.tif]
