# Supplementary material for: The Collagen Chaperone HSP47 Is a New Interactor of APP that Affects the Levels of Extracellular Beta-Amyloid Peptides
Source: PLoS One. 2011 Jul 28;6(7):e22370. doi: 10.1371/journal.pone.0022370 (PMC3145648; doi:10.1371/journal.pone.0022370)
Supplement: Table S1 — List of candidate APP partners identified by the coexpression-based bioinformatic screen. List of the 137 candidates identified by conserved coexpression analysis on the SMD dataset. A = colocalized with APP or affecting APP localization; B = overexpressed in AD or found in AD lesions; C = modulator of APP metabolism and of Aβ deposition; D = downstream mediator of APP or Aβ; E = APP binding partner. Asterisks indicate the genes reported to encode for APP interacting proteins in the HPRD database and the genes genetically linked to AD in the Alzgene database. The last column (N) indicates the number of APP conserved coexpression lists in which the corresponding gene was found. The genes are ranked by decreasing N. (PDF) [file pone.0022370.s007.pdf]

Table S1

Gene Name	Gene ID	Literature	HPRD	Alzgene	N
ANXA4	307				28
LIMA1	51474				23
WSB2	55884				23
TPM2	7169				21
DAG1	1605				20
F2R	2149	A [1]			20
COL4A2	1284		*		19
ECM1	1893				19
KRT18	3875				19
NXN	64359				19
TWSG1	57045				19
NBL1	4681				18
PLK2	10769				18
SERPINH1	871				18
TNKS1BP1	85456				17
AGRN	375790	B [2]			16
FAM129B	64855				16
CAV1	857	C [3]	*	*	16
CNN3	1266				16
PXDN	7837				16
FKBP10	60681				16
GBE1	2632				16
GJA1	2697	B [4]			16
IGFBP4	3487				16
MATN2	4147				16
BCAR1	9564				15
NCKAP1	10787	C [5]			15
PPIC	5480				15
PRDX5	25824				15
SLC4A2	6522				15
WBP5	51186				15
AP1S1	1174				14
APP	351				14
TMEM184B	25829				14
EMP1	2012				14
PSMD2	5708				14
SPRY4	81848				14
TGFB1I1	7041				14
CAPN2	824	B [6]			13
COL18A1	80781	B [7]			13
NR2F1	7025				13

ANXA3	306				12
CAV2	858	C [3]			12
CLU	1191	C [8]	*	*	12
COL1A2	1278		*		12
DNAJB4	11080				12
GORASP2	26003				12
ITGB1	3688	A [9]			12
KDR	3791				12
KIAA1522	57648				12
LRP1	4035	C [10]	*	*	12
LRPAP1	4043	C [11]		*	12
C14orf179	112752				12
NGFRAP1	27018	D [12]			12
PHLDB1	23187				12
RNF11	26994				12
SMTN	6525				12
COL1A1	1277				11
COL6A1	1291	D [13] B [14]			11
SH3D19	152503				11
FBLN1	2192	E [15]	*		11
HTATIP2	10553				11
IGFBP6	3489				11
MARCKS	4082	D [16]			11
MBTPS1	8720				11
NRP1	8829				11
TEAD2	8463				11
TENC1	23371				11
TPM4	7171	B [17]			11
CFL2	1073	D [18]			10
EFEMP2	30008				10
MAGED2	10916				10
MYL9	10398				10
UBTD1	80019				10
BGN	633	C [19]	*		9
BMP1	649				9
CRYAB	1410	C [20]	*		9
EGR1	1958	B [21]			9
UBE2Z	65264				9
GANAB	23193				9
LPL	4023	C [22]		*	9
NDRG1	10397				9
NUPR1	26471				9
PERP	64065				9
S100A6	6277	B [23]			9

SGK1	6446	D [24]			9
SURF4	6836				9
TAGLN	6876	D [25]			9
YWHAG	7532	B [26]			9
ANXA5	308	C [27]			8
ARMCX1	51309				8
ATP5B	506	B [28]			8
B4GALT4	8702				8
CASKIN2	57513				8
CD151	977				8
CDC42EP1	11135				8
CLIC1	1192	D [29]			8
COL3A1	1281				8
COL6A2	1292	D [13]			8
COL6A3	1293				8
CST3	1471	C [30]		*	8
CTSL2	1515				8
PSMG1	8624				8
FHL1	2273				8
FHL2	2274	C [31]			8
FSTL1	11167				8
H1F0	3005				8
HCFC1R1	54985				8
HEBP1	50865				8
KDELC2	143888				8
LGALS1	3956				8
LXN	56925				8
MEA1	4201				8
NEU1	4758				8
NID1	4811		*		8
PJA1	64219				8
PPAP2C	8612				8
PTMS	5763				8
SERPINF1	5176	B [32]			8
TCEAL8	90843				8
CACNG1	786				7
CBR1	873				7
HMOX2	3163	D [33]	*	*	7
ITGA5	3678				7
ITGAV	3685				7
ITGB5	3693				7
LAMP1	3916	B [34]			7
MRLC2	103910				7
NPR2	4882				7

PDGFRA	5156	C [35]			7
RCN1	5954				7
SH3BP4	23677				7
SLC25A4	291				7
SPG21	51324				7
TNFRSF12A	51330				7
TTC8	123016				7
ZIC1	7545				7

.

Supplemental references

1. Brewer GJ (1996) Thrombin causes cell spreading and redistribution of beta-amyloid immunoreactivity in cultured hippocampal neurons. J Neurochem 67: 119-130.
2. Verbeek MM, Otte-Holler I, van den Born J, van den Heuvel LP, David G, et al. (1999) Agrin is a major heparan sulfate proteoglycan accumulating in Alzheimer's disease brain. Am J Pathol 155: 2115-2125.
3. van Helmond ZK, Miners JS, Bednall E, Chalmers KA, Zhang Y, et al. (2007) Caveolin-1 and -2 and their relationship to cerebral amyloid angiopathy in Alzheimer's disease. Neuropathol Appl Neurobiol 33: 317-327.
4. Nagy JI, Li W, Hertzberg EL, Marotta CA (1996) Elevated connexin43 immunoreactivity at sites of amyloid plaques in Alzheimer's disease. Brain Res 717: 173-178.
5. Yamamoto A, Behl C (2001) Human Nck-associated protein 1 and its binding protein affect the metabolism of beta-amyloid precursor protein with Swedish mutation. Neurosci Lett 316: 50-54.
6. Adamec E, Mohan P, Vonsattel JP, Nixon RA (2002) Calpain activation in neurodegenerative diseases: confocal immunofluorescence study with antibodies specifically recognizing the active form of calpain 2. Acta Neuropathol 104: 92-104.
7. van Horssen J, Wilhelmus MM, Heljasvaara R, Pihlajaniemi T, Wesseling P, et al. (2002) Collagen XVIII: a novel heparan sulfate proteoglycan associated with

vascular amyloid depositions and senile plaques in Alzheimer's disease brains. Brain Pathol 12: 456-462.
8. Holtzman DM (2004) In vivo effects of ApoE and clusterin on amyloid-beta metabolism and neuropathology. J Mol Neurosci 23: 247-254.
9. Storey E, Beyreuther K, Masters CL (1996) Alzheimer's disease amyloid precursor protein on the surface of cortical neurons in primary culture co-localizes with adhesion patch components. Brain Res 735: 217-231.
10. Liu Q, Zerbinatti CV, Zhang J, Hoe HS, Wang B, et al. (2007) Amyloid precursor protein regulates brain apolipoprotein E and cholesterol metabolism through lipoprotein receptor LRP1. Neuron 56: 66-78.
11. Sanchez L, Alvarez V, Gonzalez P, Gonzalez I, Alvarez R, et al. (2001) Variation in the LRP-associated protein gene (LRPAP1) is associated with late-onset Alzheimer disease. Am J Med Genet 105: 76-78.
12. Podlesniy P, Kichev A, Pedraza C, Saurat J, Encinas M, et al. (2006) Pro-NGF from Alzheimer's disease and normal human brain displays distinctive abilities to induce processing and nuclear translocation of intracellular domain of p75NTR and apoptosis. Am J Pathol 169: 119-131.
13. Cheng JS, Dubal DB, Kim DH, Legleiter J, Cheng IH, et al. (2009) Collagen VI protects neurons against Abeta toxicity. Nat Neurosci 12: 119-121.
14. Cullen KM, Kocsi Z, Stone J (2006) Microvascular pathology in the aging human brain: evidence that senile plaques are sites of microhaemorrhages. Neurobiol Aging 27: 1786-1796.
15. Ohsawa I, Takamura C, Kohsaka S (2001) Fibulin-1 binds the amino-terminal head of beta-amyloid precursor protein and modulates its physiological function. J Neurochem 76: 1411-1420.

16. Murphy A, Sunohara JR, Sundaram M, Ridgway ND, McMaster CR, et al. (2003) Induction of protein kinase C substrates, Myristoylated alanine-rich C kinase substrate (MARCKS) and MARCKS-related protein (MRP), by amyloid beta-protein in mouse BV-2 microglial cells. Neurosci Lett 347: 9-12.

17. Galloway PG, Perry G (1991) Tropomyosin distinguishes Lewy bodies of Parkinson disease from other neurofibrillary pathology. Brain Res 541: 347-349.

18. Maloney MT, Bamburg JR (2007) Cofilin-mediated neurodegeneration in Alzheimer's disease and other amyloidopathies. Mol Neurobiol 35: 21-44.

19. Bjelik A, Pakaski M, Bereczki E, Gonda S, Juhasz A, et al. (2007) APP mRNA splicing is upregulated in the brain of biglycan transgenic mice. Neurochem Int 50: 1-4.

20. Wilhelmus MM, Boelens WC, Otte-Holler I, Kamps B, de Waal RM, et al. (2006) Small heat shock proteins inhibit amyloid-beta protein aggregation and cerebrovascular amyloid-beta protein toxicity. Brain Res 1089: 67-78.

21. Rensink AA, Gellekink H, Otte-Holler I, ten Donkelaar HJ, de Waal RM, et al. (2002) Expression of the cytokine leukemia inhibitory factor and pro-apoptotic insulin-like growth factor binding protein-3 in Alzheimer's disease. Acta Neuropathol 104: 525-533.

22. Blain JF, Aumont N, Theroux L, Dea D, Poirier J (2006) A polymorphism in lipoprotein lipase affects the severity of Alzheimer's disease pathophysiology. Eur J Neurosci 24: 1245-1251.

23. Boom A, Pochet R, Authelet M, Pradier L, Borghgraef P, et al. (2004) Astrocytic calcium/zinc binding protein S100A6 over expression in Alzheimer's disease and in PS1/APP transgenic mice models. Biochim Biophys Acta 1742: 161-168.

24. Lee EJ, Chun J, Hyun S, Ahn HR, Jeong JM, et al. (2008) Regulation Fe65 localization to the nucleus by SGK1 phosphorylation of its Ser566 residue. BMB Rep 41: 41-47.

25. Muller T, Concannon CG, Ward MW, Walsh CM, Tirniceriu AL, et al. (2007) Modulation of gene expression and cytoskeletal dynamics by the amyloid precursor protein intracellular domain (AICD). Mol Biol Cell 18: 201-210.
26. Sugimori K, Kobayashi K, Kitamura T, Sudo S, Koshino Y (2007) 14-3-3 protein beta isoform is associated with 3-repeat tau neurofibrillary tangles in Alzheimer's disease. Psychiatry Clin Neurosci 61: 159-167.
27. Lee G, Pollard HB, Arispe N (2002) Annexin 5 and apolipoprotein E2 protect against Alzheimer's amyloid-beta-peptide cytotoxicity by competitive inhibition at a common phosphatidylserine interaction site. Peptides 23: 1249-1263.
28. Shin SJ, Lee SE, Boo JH, Kim M, Yoon YD, et al. (2004) Profiling proteins related to amyloid deposited brain of Tg2576 mice. Proteomics 4: 3359-3368.
29. Novarino G, Fabrizi C, Tonini R, Denti MA, Malchiodi-Albedi F, et al. (2004) Involvement of the intracellular ion channel CLIC1 in microglia-mediated beta-amyloid-induced neurotoxicity. J Neurosci 24: 5322-5330.
30. Kaeser SA, Herzig MC, Coomaraswamy J, Kilger E, Selenica ML, et al. (2007) Cystatin C modulates cerebral beta-amyloidosis. Nat Genet 39: 1437-1439.
31. Tanahashi H, Tabira T (2000) Alzheimer's disease-associated presenilin 2 interacts with DRAL, an LIM-domain protein. Hum Mol Genet 9: 2281-2289.
32. Yamagishi S, Inagaki Y, Takeuchi M, Sasaki N (2004) Is pigment epithelium-derived factor level in cerebrospinal fluid a promising biomarker for early diagnosis of Alzheimer's disease? Med Hypotheses 63: 115-117.
33. Takahashi M, Dore S, Ferris CD, Tomita T, Sawa A, et al. (2000) Amyloid precursor proteins inhibit heme oxygenase activity and augment neurotoxicity in Alzheimer's disease. Neuron 28: 461-473.

34. Barrachina M, Maes T, Buesa C, Ferrer I (2006) Lysosome-associated membrane protein 1 (LAMP-1) in Alzheimer's disease. Neuropathol Appl Neurobiol 32: 505-516.

35. Gianni D, Zambrano N, Bimonte M, Minopoli G, Mercken L, et al. (2003) Platelet-derived growth factor induces the beta-gamma-secretase-mediated cleavage of Alzheimer's amyloid precursor protein through a Src-Rac-dependent pathway. J Biol Chem 278: 9290-9297.
